# Splenic-targeting biomimetic nanovaccine for elevating protective immunity against virus infection

**DOI:** 10.1186/s12951-022-01730-0

**Published:** 2022-12-03

**Authors:** Jian Huo, Angke Zhang, Shuqi Wang, Hanghang Cheng, Daopeng Fan, Ran Huang, Yanan Wang, Bo Wan, Gaiping Zhang, Hua He

**Affiliations:** College of Veterinary Medicine, International Joint Research Center of National Animal Immunology, Henan Engineering Laboratory of Animal Biological Products, Longhu Laboratory, Henan Agricultural University, Zhengzhou, 450046 China

**Keywords:** Red blood cell membrane, Targeting, Biomimetic nanovaccine, CpG, ASFV

## Abstract

**Background:**

The prevalence of viral infectious diseases has become a serious threat to public safety, economic and social development. Vaccines have been served as the most effective platform to prevent virus transmission via the activation of host immune responses, while the low immunogenicity or safety, the high cost of production, storage, transport limit their effective clinical application. Therefore, there is a need to develop a promising strategy to improve the immunogenicity and safety of vaccines.

**Methods:**

We developed a splenic-targeting biomimetic nanovaccine (NV) that can boost protective humoral and cellular immunity against african swine fever virus (ASFV) infection. The universal PLGA nanoparticles (CMR-PLGA/p54 NPs) coated with mannose and CpG (TLR9 agonist) co-modified red blood cell (RBC) membrane were prepared, which comprised a viral antigen (p54) and can be served as a versatile nanovaccine for elevating protective immunity.

**Results:**

CMR-PLGA/p54 NVs could be effectively uptaken by BMDC and promoted BMDC maturation in vitro. After subcutaneous immunization, antigen could be effectively delivered to the splenic dendritic cells (DCs) due to the splenic homing ability of RBC and DC targeting capacity of mannose, which promoted antigen presentation and DCs maturation, and further elicited higher levels of cytokines secretion and specific IgG titers, CD4^+^ and CD8^+^ T cells activation and B maturation. Moreover, NVs demonstrated notable safety during the immunization period.

**Conclusions:**

This study demonstrates the high potential of CMR-PLGA NPs as vaccine delivery carriers to promote humoral and cellular immune responses, and it provides a promising strategy to develop safe and effective vaccines against viral infectious diseases.

**Supplementary Information:**

The online version contains supplementary material available at 10.1186/s12951-022-01730-0.

## Introduction

Viral infectious diseases, such as COVID-19, AIDS [1, 2], influenza, and African swine fever (ASF) are emerging pandemics around the world, which have seriously threatened public security, economic and social development [3–6]. Vaccines have been served as the most effective platform to prevent virus transmission via the activation of immune responses, thus reducing morbidity and mortality [7]. Currently, traditional vaccines consisted of attenuated, inactivated pathogens or recombinant proteins, are able to elicit host immune responses to protect against viral infection, while the low immunogenicity or safety limit their effective clinical application [8, 9]. For instance, multiple and high dose and adjuvants assistance have usually been required during vaccination, which are attributed to the low immunogenicity of inactivated vaccines [10]. Aluminum salt adjuvant is one of the most common adjuvants, however, it can produce limited cellular immune response and side effect (swelling, fever, neurotoxicity, etc.) [11]. Besides, the high cost of production, storage, transport, and in vivo degradation and minimal accumulation at desired sites are major obstacles that restrict wide and effective application of vaccines [12]. Therefore, a promising strategy need to be developed to improve the immunogenicity and safety of vaccines.

As an emerging manner, nanocarriers such as inorganic nanoparticles (NPs), polymeric NPs, liposomes have shown remarkable potential in antigen delivery system and immunostimulatory adjuvants [13–15]. Nanocarriers can be engineered to possess numerous features [16], such as sustainable release of antigen during a long period, preventing antigen degradation [17, 18], and co-encapsulation of antigen and adjuvants [19, 20]. Moreover, the nanoscale size and surface charge of nanocarriers contribute to antigen accumulation in lymph nodes (LNs) [21, 22], resulting in achieving systemic transportation of antigen and eliciting protective immune responses [23]. Alternatively, nanocarriers are capable of cytosolic antigens delivery to achieve antigen cross-presentation [24], which can elicit potent T cell responses to realize disease prevention and treatment [25, 26]. However, some nanocarriers can be eliminated by the mononuclear phagocyte system before uptake by the antigen-presenting cells (APCs) at the tissue site, thus hampering antigen presentation to lymphocytes. Hence, it is desired that antigen should be effectively and specifically delivered to APCs to boost protective immunity [27].

The targeting antigen delivery that can be developed by decorating NPs with ligands or antibodies specific to APCs, has been an effective approach to enhance vaccines immune responses [28, 29]. The mannose receptor (MR), a calcium-dependent lectin overexpressed on the surfaces of APCs, such as DCs and macrophages [30, 31]. Targeting delivery of antigens to MR on DCs can improve antigen uptake, presentation, and regulate the APCs differentiation and maturation [32–34]. Recently, cell membrane coating has been proposed to be an emerging manner for NPs to enhance biointerfacing capacities [35–37]. Based on convenient preparation and remarkable biocompatibility, red blood cells (RBCs) can be used to prolong the NPs circulation time [38]. Besides, spleen is a critical lymphoid organs that contains abundant APCs, B cells and T cells [39]. When the RBCs have been damaged or aged, RBCs are phagocytosed by scavenging cells, including spleen-resident DCs and macrophages. Therefore, damaged RBCs membrane may have the ability to “target” the spleen [40–42].

**Scheme 1:**
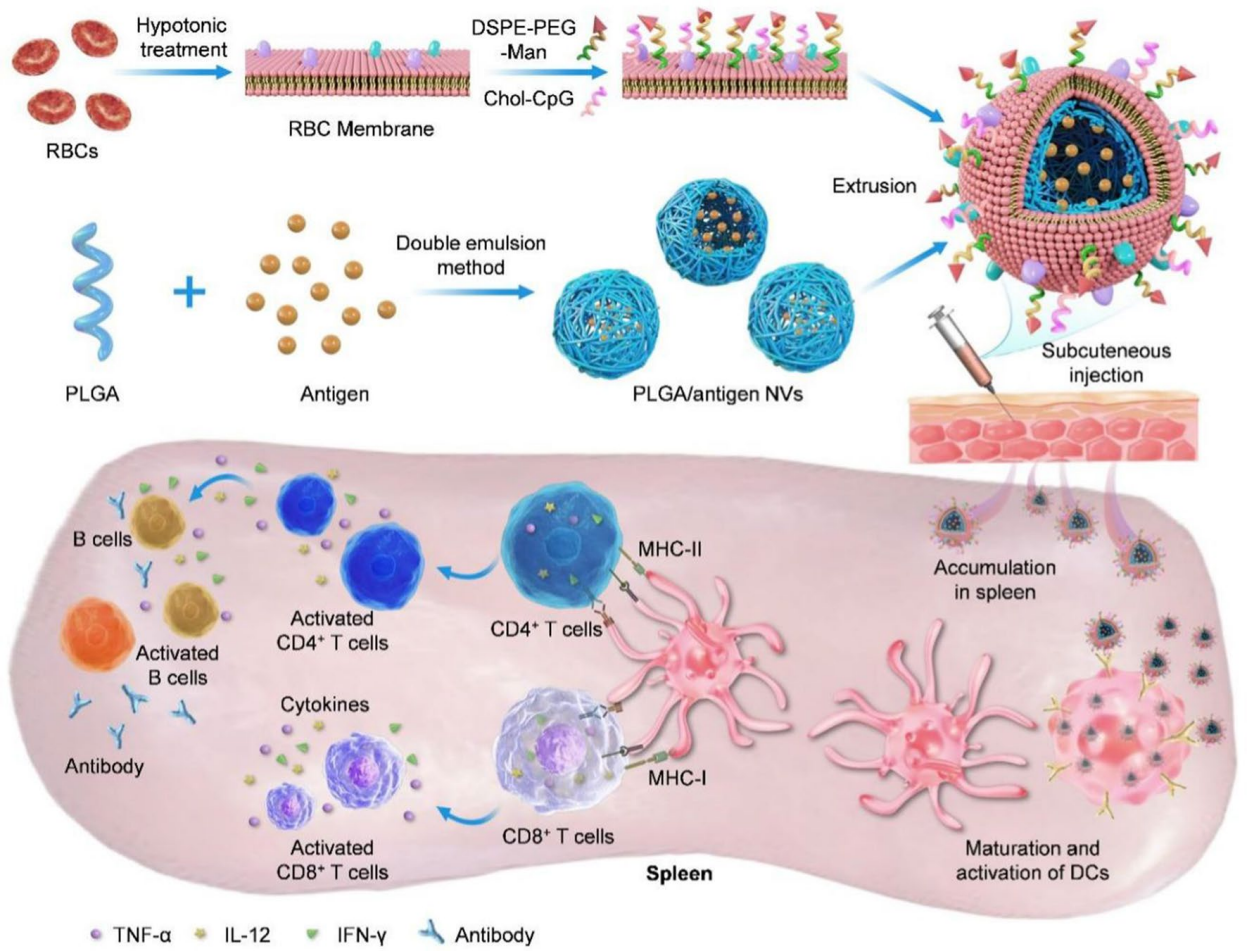
Schematic illustration of splenic targeting nanovaccine for combating virus infection. Viral antigen (p54) loaded PLGA nanovaccine were prepared via double-emulsion, which was further coated with CpG and mannose co-modified RBCs membrane to improve the serum stability and splenic targeting. After subcutaneous immunization, nanovaccine effectively delivered antigen to the splenic dendritic cells (DCs), improved the DCs maturation, antigen presentation, cytokines secretion, antibody production, B cells maturation, and T cells activation, thus achieving a robust humoral and cellular immunity

In this study, we report a DC-targeting antigen delivery system, which features the loading capacity of PLGA NPs and natural property of RBCs membrane, attempting to boost a protective humoral and cellular immunity (Scheme 1). To this end, the african swine fever virus (ASFV) antigen (p54) loaded PLGA nanovaccines (NVs) were prepared via double-emulsion, and further coated with RBCs membrane to improve the serum stability and splenic targeting of PLGA NPs, thus achieving long circulation in vivo and spleen accumulation. DSPE-PEG-mannose (DSPE-PEG-Man) and chol-CpG (a TLR9 agonist [43, 44]) were then incorporated into RBCs membrane to formulate CpG and mannose-modified PLGA (CMR-PLGA, C represents CpG, M represents mannose, R represents RBCs membrane) NPs, which could be able to actively target DCs in the spleen by the affinity between mannose and mannose receptor overexpressed on DC membrane. After subcutaneous injection, CMR-PLGA/p54 NVs could positively accumulate in the spleen, and then actively integrate with DCs via MR, promote antigen presentation, stimulate the DCs maturation, and subsequently elicit high level of cytokines and anti-p54 antibodies, T cells activation, and B maturation, thus achieving an efficient humoral and cellular immunity. In conclusion, CMR-PLGA/p54 NVs would be an effective versatile vaccine, which renders a promising application in the prevention of viral infectious diseases.

## Methods

### The preparation of CMR-PLGA/OVA NVs

PLGA/OVA nanovaccines (NVs) were prepared by the double emulsion (w/o/w) method as described previously [10]. OVA and PLGA were separately dissolved in DCM (5 mg/mL, 1 mL) and 1% w/v PVA solution (3 mg/mL, 0.2 mL), which were mixed and sonicated (20,000 J, 2 min) under ice-water bath to form w/o emulsion. Then the w/o emulsion was emulsified with 2% w/v PVA solution (3 mL, 50,000 J, 10 min) under ice-water bath to form w/o/w double emulsion, which was further added to 2% w/v PVA solution (3 mL), stirred at room temperature (RT) overnight to evaporate DCM. Finally, the mixture was centrifuged (6100 rpm, 20 min) to collect the supernatant, thus obtaining PLGA/OVA NVs. To prepare the RBC membrane coated PLGA/OVA NVs, the CpG-Man-RBCm (5 mg) was mixed with PLGA/OVA NVs (0.8 mg/mL) and the mixture further extruded by the Avanti Polar Lipids to yeild RBC membrane coated PLGA/OVA NVs (CMR-PLGA/OVA NVs).

### Cellular uptake and intracellular distribution

BMDCs were seeded on 24-well plates at 1 × 10^6^ cells/well and cultured for 2 h. The cells were treated with free FITC/OVA or MR-PLGA/FITC-OVA NVs (0.02 mg FITC-OVA/mL) for 12 h. The cells were then stained with DAPI (5 µg/mL) before confocal laser scanning microscope (CLSM) observations. To further evaluate the mannose-assisted targeting effect, BMDCs were pretreated with mannose (10 mg/mL) for 2 h, washed with PBS before MR-PLGA/FITC-OVA NVs were added.

To assess the cellular uptake of NVs, BMDCs were incubated with MR-PLGA/FITC-OVA NVs for 12 h, and further lysed with RIPA lysis buffer. Protein and FITC content in the mixture were measured by BCA kit and spectrofluorimetry (λ_ex_ = 490 nm, λ_em_ = 525 nm), respectively. To further investigate the mannose-mediated targeting effect, cells were pretreated with mannose (10 mg/mL) for 2 h, washed with PBS before MR-PLGA/FITC-OVA NVs were added.

### Activation and maturation of BMDCs

To estimate the effect of NVs on DCs maturation, bone marrow-derived dendritic cells (BMDCs) were incubated with various NVs or free OVA (0.02 mg OVA/mL) for 12 h. The collected BMDCs were stained with anti-CD11c-APC, anti-CD86-FITC, anti-CD80-FITC, anti-SIINFEKL-PE, anti-MHC-II-FITC for 30 min before measurement by flow cytometry. Moreover, the concentration of IFN-γ, TNF-α, and IL-12 in the culture supernatant was quantified with ELISA kits according to the protocol.

### Animal Immunization and immune responses in vivo

ASFV protein p54 encapsulated NVs (CMR-PLGA/p54) were prepared using the same methods as described above. BALB/c mice were randomly divided into 6 groups (n = 14), and subcutaneously immunized with free p54, PBS, p54 + Freund's adjuvant (FA, 50 μL) mixture, PLGA/p54 NVs, MR-PLGA/p54 NVs, CMR-PLGA/p54 NVs on day 0, 14, 28, 35 (30 μg p54/mouse). To evaluate the DC maturation, the spleen was harvested from immunized mice on different time points, homogenized, filtered with 70 μm sieve to obtain single-cell suspensions. The suspensions were lysed with the red blood cell lysate for 2–3 min, washed with PBS, stained with anti-CD11-APC, anti-CD80-FITC, anti-CD86-FITC before flow cytometry analysis. Besides, spleen or LNs was homogenized in RIPA lysis buffer, and centrifuged (15,000 rpm, 4 ℃) for 20 min. The content of IL-12, TNF-α, IFN-γ cytokines in supernatant was quantified by ELISA kits.

To investigate the proliferation of splenocytes, the splenic single-cell was retreated with NVs (2 μg/well) as described above in the animal immunization assay. After re-stimulation for 12 h, the cells were counted to evaluate splenocytes proliferation, and the culture medium was collected to detect IFN-γ content via ELISA kit. Moreover, to evaluate T cell responses, the cells were stained with anti-CD4-FITC, anti-CD3-APC, anti-CD8-FITC for 1 h before flow cytometry analysis. To evaluate maturation of B cells, the cells were stained with anti-CD19-APC, anti-IgD-FITC for 1 h before flow cytometry analysis.

To evaluate the IgG level, blood was collected from immunized mice on day 14, 28, 35, 49, and further centrifuged (3500 rpm, 4 °C) for 20 min to collect serum. IgG, IgG1, IgG2a content and p54 antibody titers in serum were determined by ELISA kits. Besides, the major tissues were harvested from immunized mice on day 49, subjected to histological examination by H&E staining and immunohistochemical staining.

## Results and discussion

### Preparation and characterization of CMR-PLGA/OVA NVs

**Fig. 1:**
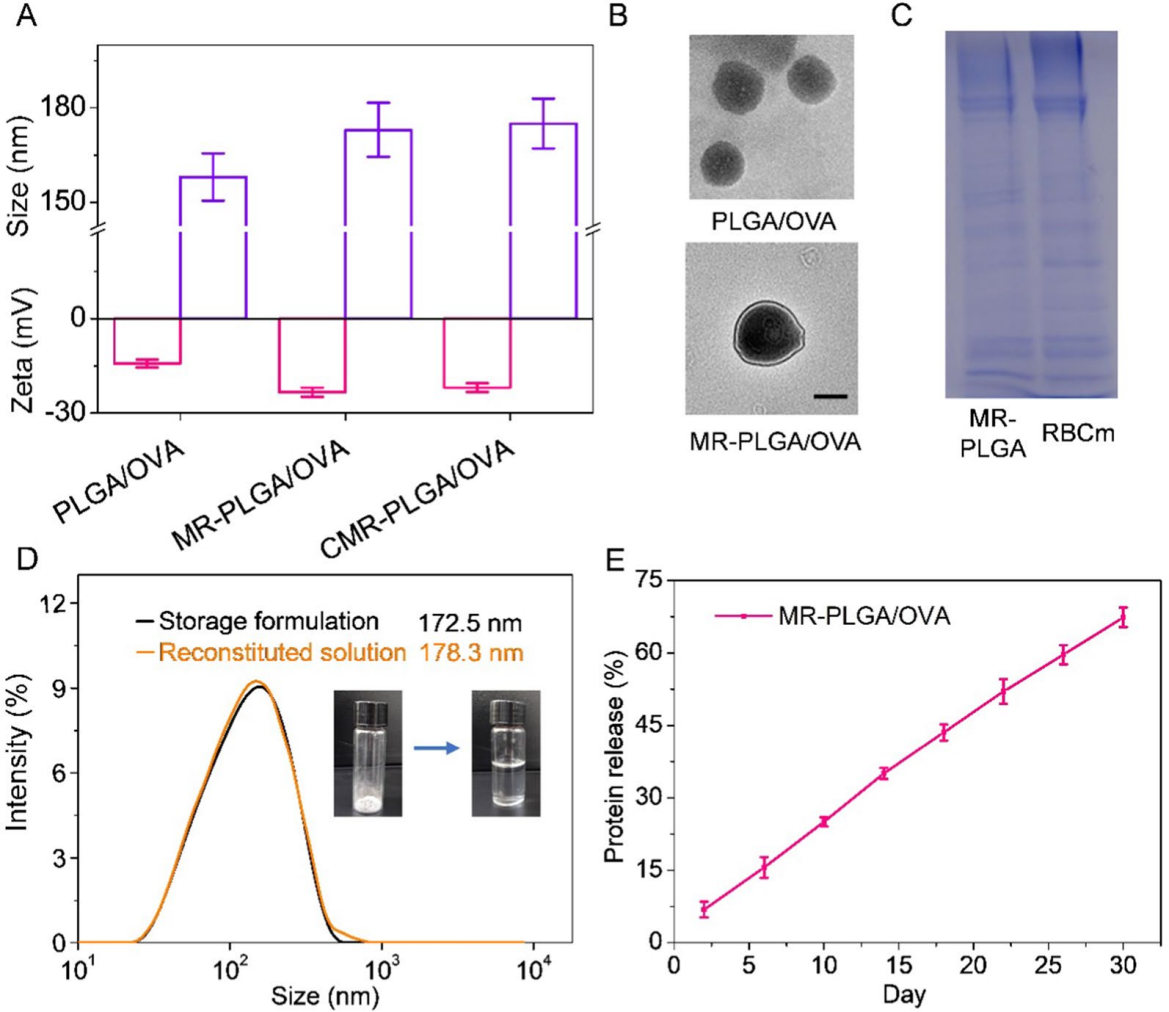
Characterization of MR-PLGA/OVA NVs. **A** Size and zeta potential of PLGA NVs, MR-PLGA/OVA NVs and CMR-PLGA/OVA NVs (n = 3). **B** Representative TEM images of PLGA NVs and MR-PLGA/OVA NVs. The scale bar represents 100 nm. **C** SDS-PAGE pattern of proteins from RBC membrane and MR-PLGA/OVA NVs. **D** The particle size of MR-PLGA/OVA NVs before and after lypophilization. **E** In vitro OVA release profile from MR-PLGA/OVA NVs (n = 3)

To targeting delivery of antigens, PLGA/OVA NVs were fabricated via the double emulsion process, and the CpG-Man-RBC membrane was further coated on the surfaces of PLGA/OVA NVs. As shown in Fig. 1A, PLGA/OVA NVs showed the particle size of ≈ 158 nm and the zeta potential of − 14.2 mV. After negatively charged RBC membrane coating, the particle size of MR-PLGA/OVA NVs increased to ≈ 173 nm and the zeta potential decreased to − 23.3 mV. TEM images also demonstrated that MR-PLGA/OVA NVs possessed the spherical core–shell morphology with the particle size of ≈ 160 nm, consisting with the DLS measurement (Fig. 1B). Besides, the particle size of MR-PLGA/OVA NVs had minimal alteration after incubation with serum or PBS during 7 days, suggesting that the NVs had ideal stability (Additional file 1: Fig. S1).

Alternatively, the SDS-PAGE demonstrated that MR-PLGA NPs had the similar protein expression as RBC membrane, which suggested that the RBC membrane was successfully coated onto PLGA NPs (Fig. 1C). After lyophilization and re-dissolution in PBS, the particle size of MR-PLGA/OVA NVs negligibly altered (Fig. 1D). The PLC and PLE of OVA was 8.1% and 75%, respectively. The in vitro OVA release from MR-PLGA/OVA NVs was further investigated. As shown in Fig. 1E, sustained OVA release was noted, achieving a cumulative amount of ~ 67.4% within 30 days.

### Mannose-mediated BMDCs targeting and cellular uptake

**Fig. 2:**
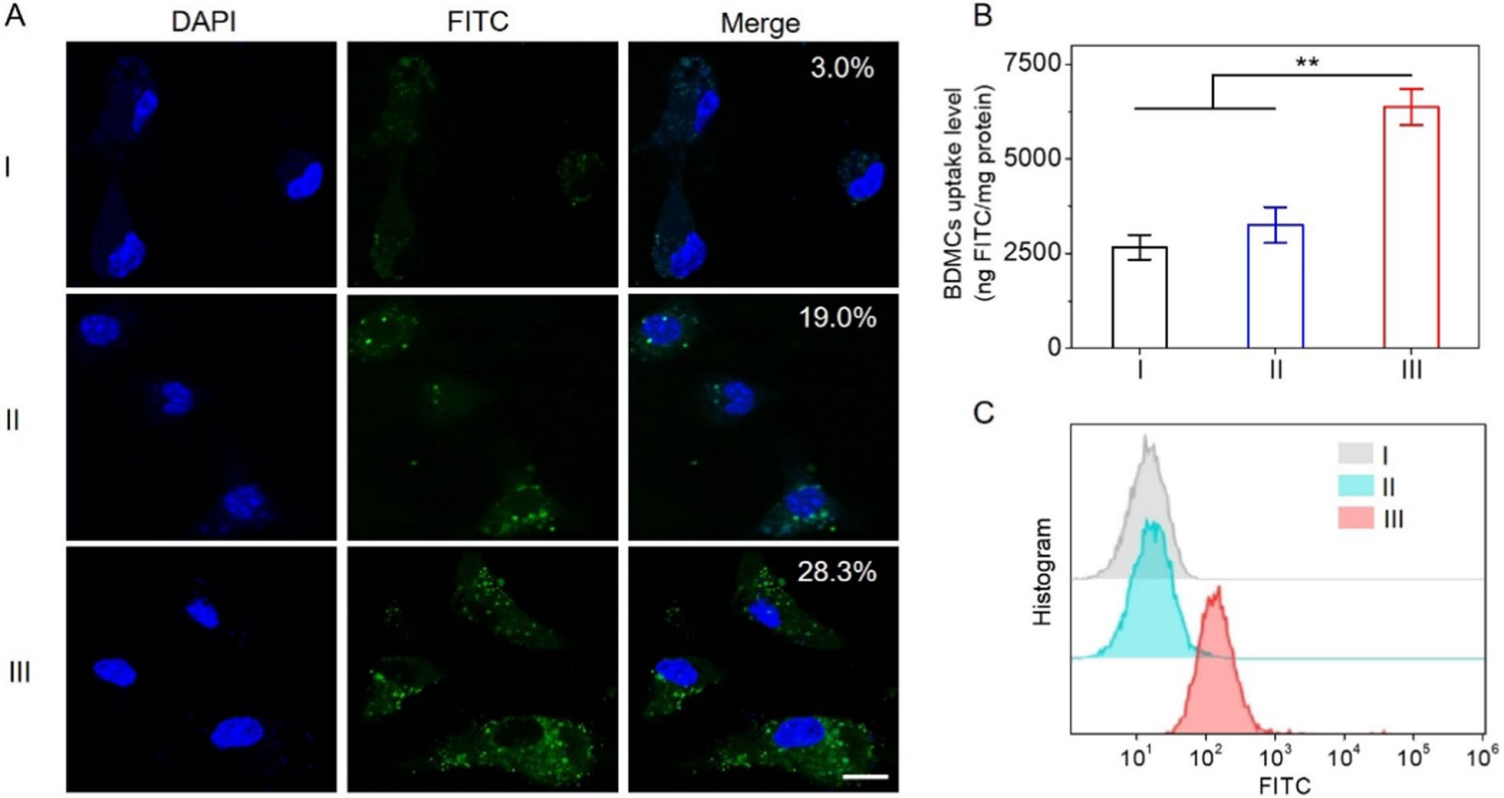
MR-PLGA/OVA NVs mediated targeted delivery of antigen to BMDCs in vitro. **A** CLSM images of BMDCs following incubation with free OVA, or MR-PLGA/OVA NVs for 12 h. BMDCs were pretreated with mannose for 2 h to block mannose receptor. The scale bar represents 10 μm. **B** Uptake level of MR-PLGA/OVA NVs with or without mannose pretreatment (n = 3). **C** Uptake of free OVA and MR-PLGA/OVA NVs in BMDCs as assessed by flow cytometry. The BMDCs were pretreated as described in (**A**). (I: free FITC-OVA; II: MR-PLGA/FITC-OVA NVs (w/ Man); III: MR-PLGA/FITC-OVA NVs.)

Cellular uptake is the critical step for antigen delivery systems. CLSM images demonstrated that free FITC-OVA was negligibly taken up by BMDCs because of its hydrophilicity and high molecular weight (Fig. 2A). In comparison, green fluorescence was distributed in the cytoplasm when the FITC-OVA was loaded in MR-PLGA NVs, suggesting notable internalization of OVA assisted by MR-PLGA NVs. Pretreatment of BMDCs with mannose led to greatly reduced cytoplasmic distribution of green fluorescence, indicating mannose-mediated BMDCs targeting via recognition of mannose receptor. The cellular uptake level was further evaluated via spectrofluorimetry and flow cytometry (Fig. 2B, C). In consistence with CLSM observation, MR-PLGA/OVA NVs had higher cell uptake levels, while remarkable decrease of cellular uptake level was demonstrated under mannose pretreatment. These results collectively suggested that MR-PLGA/OVA NVs delivered OVA into BMDCs via mannose-mediated endocytosis.

### In vitro MR-PLGA/OVA NVs-elicited BMDCs maturation

**Fig. 3:**
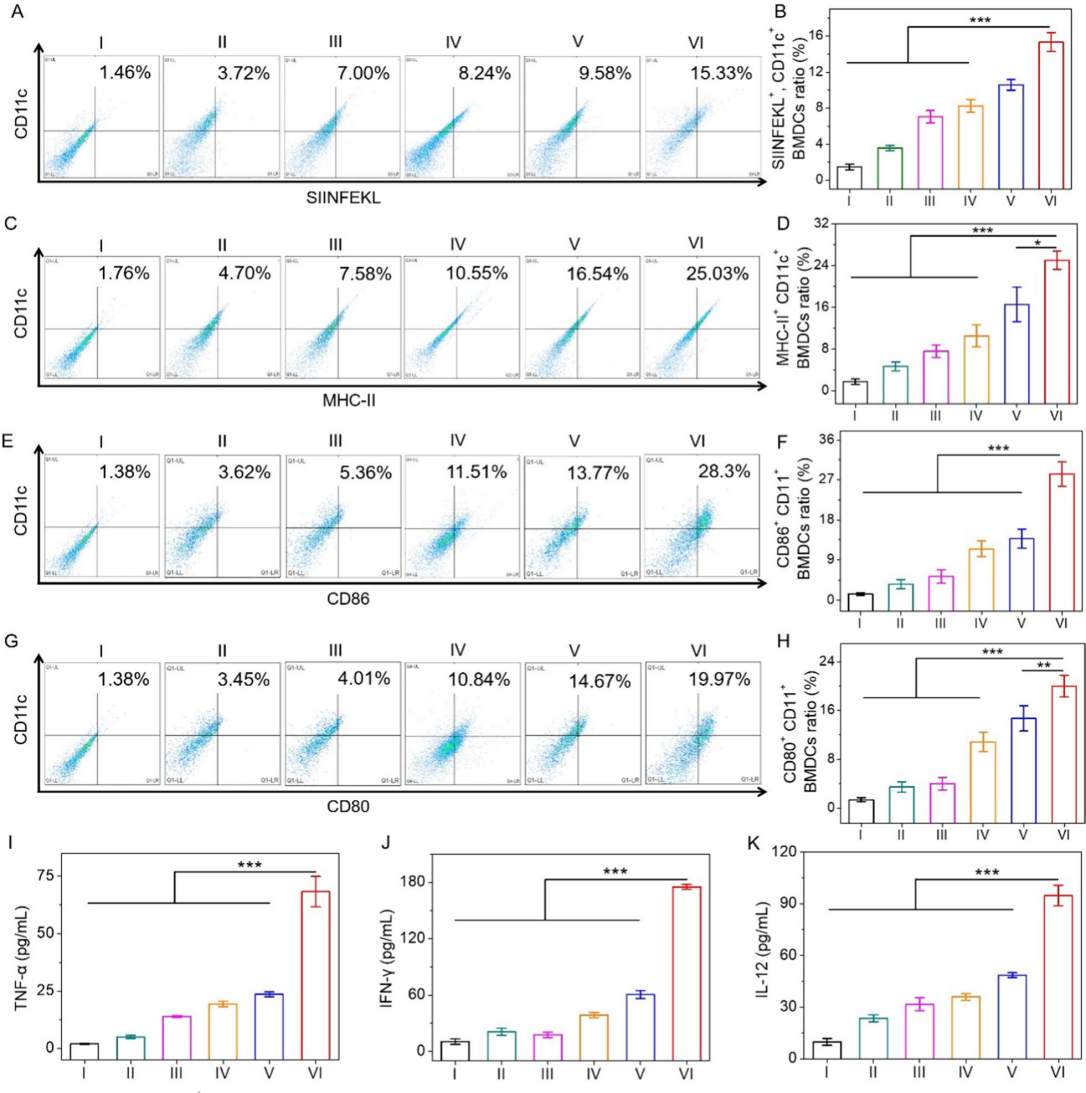
CMR-PLGA/OVA NVs-elicited BMDCs maturation in vitro. Flow cytometry (**A**, **C**) and statistic (**B**, **D**) analysis of antigen presentation in BMDCs following incubation with different groups for 12 h. Flow cytometry (**E**, **G**) and statistic (**F**, **H**) analysis of matured BMDCs following incubation with different groups for 12 h (n = 3). (**I**–**K**) The secretion level of TNF-α, IFN-γ and IL-12 from matured BMDCs treated as described in (**A**) (n = 3). (I: PBS; II: free OVA; III: PLGA/OVA NVs; IV: R-PLGA/OVA NVs; V: MR-PLGA/OVA NVs; VI: CMR-PLGA/OVA NVs.)

The process that NVs elicited antigen presentation and BMDCs maturation was then explored using flow cytometry and ELISA assay. Mature DCs overexpress the co-stimulatory molecules, MHC-I, MHC-II on the cell surfaces, including CD80 and CD86, along with cytokine production [45]. Moreover, SIINFEKL (OVA_257-264_ peptide) is able to complex with MHC-I for cross-priming CD8^+^ T cells. To explore the antigen presentation and BMDCs maturation, PBS, free OVA, PLGA/OVA NVs, R-PLGA/OVA NVs, MR-PLGA/OVA NVs, CMR-PLGA/OVA NVs were separately incubated with BMDCs for 12 h. Compared to free OVA or R-PLGA/OVA NVs, higher percentage of SIINFEKL^+^ CD11c^+^, MHC-II^+^ CD11c^+^ DCs were demonstrated after treatment with MR-PLGA/OVA NVs (Fig. 3A–D), indicating that mannose modification contributed to antigen presentation. Excitingly, CMR-PLGA/OVA NVs-treated BMDCs exhibited the maximum proportion of SIINFEKL^+^ CD11c^+^, CD80^+^ CD11c^+^, CD86^+^ CD11c^+^, MHC-II^+^ CD11c^+^, suggesting that CMR-PLGA/OVA NVs could effectively provoke antigen presentation and BMDCs maturation (Fig. 3A–H). In consistence with flow cytometry, the levels of cytokines in the supernatant collected from BMDCs were further revealed the similar results. IL-12 and TNF-α play an important role in stimulating T cell proliferation and eliciting the protective cellular immune responses [46–48]. As illustrated in Fig. 3I–K, significantly higher levels of IL-12, TNF-α, IFN-γ were found in BMDCs treated with CMR-PLGA/OVA NVs. These results collectively indicated that CMR-PLGA/OVA NVs could effectively provoke antigen presentation and the maturation of BMDCs in vitro.

### In vivo biodistribution

**Fig. 4:**
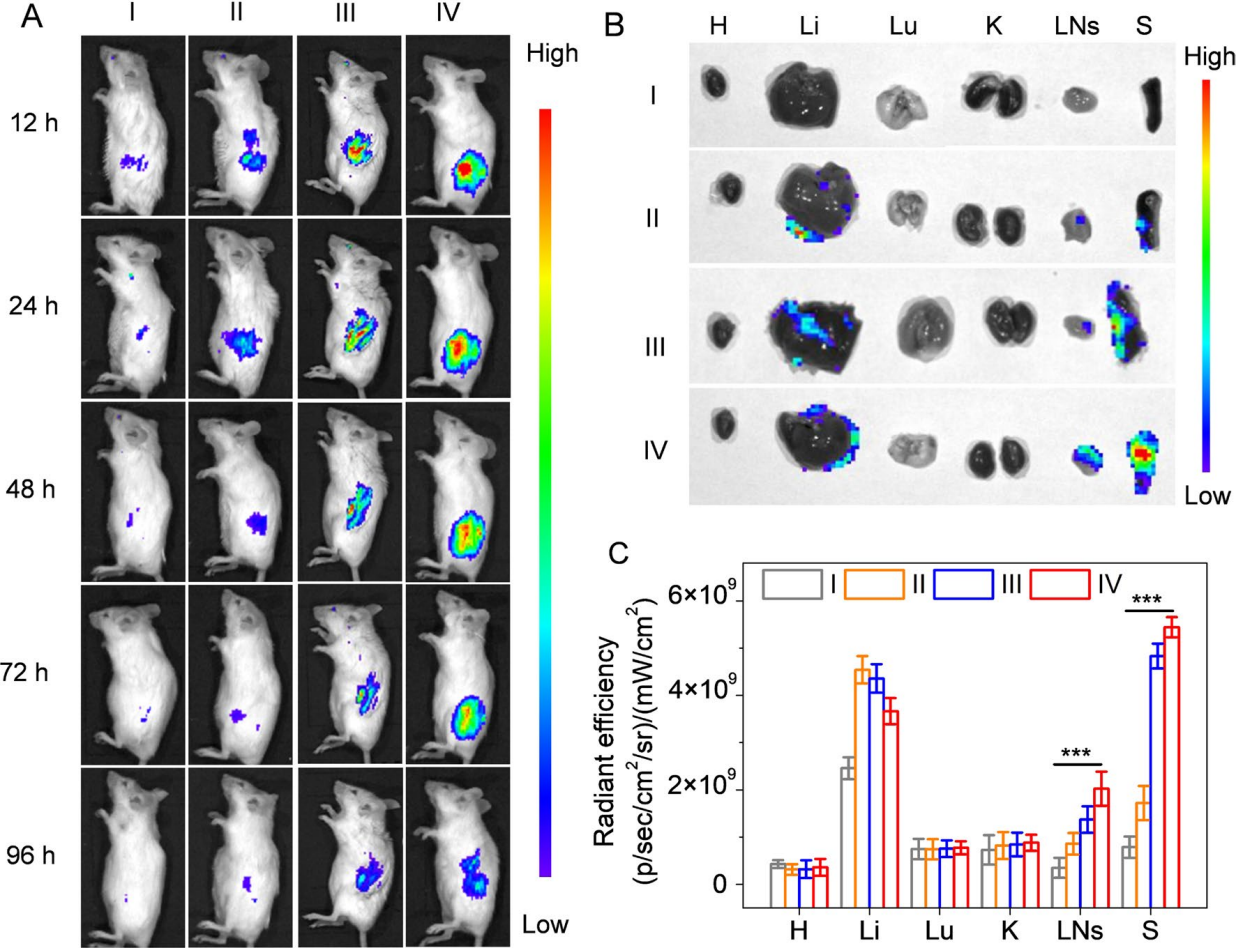
Biodistribution of MR-PLGA/OVA NVs in vivo. **A** In vivo fluorescence imaging of mice injected with different groups. **B** Ex vivo fluorescence imaging of major organs at 72 h post subcutaneous injection. (H: Heart; Li: liver; Lu: Lung; K: Kidney; LNs: Lymph nodes; S: Spleen) **C** Biodistribution level of Cy5.5-OVA in major organs at 72 h post subcutaneous injection (n = 3). (I: free Cy5.5-OVA; II: PLGA/Cy5.5-OVA NVs; III: R-PLGA/Cy5.5-OVA NVs; IV: MR-PLGA/Cy5.5-OVA NVs.)

To observe the in vivo distribution, the free Cy5.5-OVA, PLGA/Cy5.5-OVA NVs, R-PLGA/Cy5.5-OVA NVs and MR-PLGA/Cy5.5-OVA NVs were injected to mice. Compared to PLGA/Cy5.5-OVA NVs, the enhanced fluorescence signal of Cy5.5 was demonstrated in R-PLGA/Cy5.5-OVA NVs-treated mice (Fig. 4A, B), which was attributed to the splenic targeting ability of RBCs membrane. Excitingly, the Cy5.5 fluorescence in MR-PLGA/Cy5.5-OVA NVs-treated mice was stronger than that in other groups-treated mice at 72 h post injection. Moreover, the Cy5.5 fluorescence of MR-PLGA/Cy5.5-OVA NVs was still strong in the spleen even at 96 h post injection. The harvested tissues from immunized mice were further subjected to ex vivo imaging and quantitative analysis (Fig. 4B, C). Consistence with in vivo fluorescence imaging, the spleen accumulation level of Cy5.5 was higher than free Cy5.5-OVA and PLGA/Cy5.5-OVA NVs, which suggested that MR-PLGA/OVA NVs possessed the high spleen accumulation and retention.

### In vivo CMR-PLGA/p54 NVs-elicited DCs maturation

**Fig. 5:**
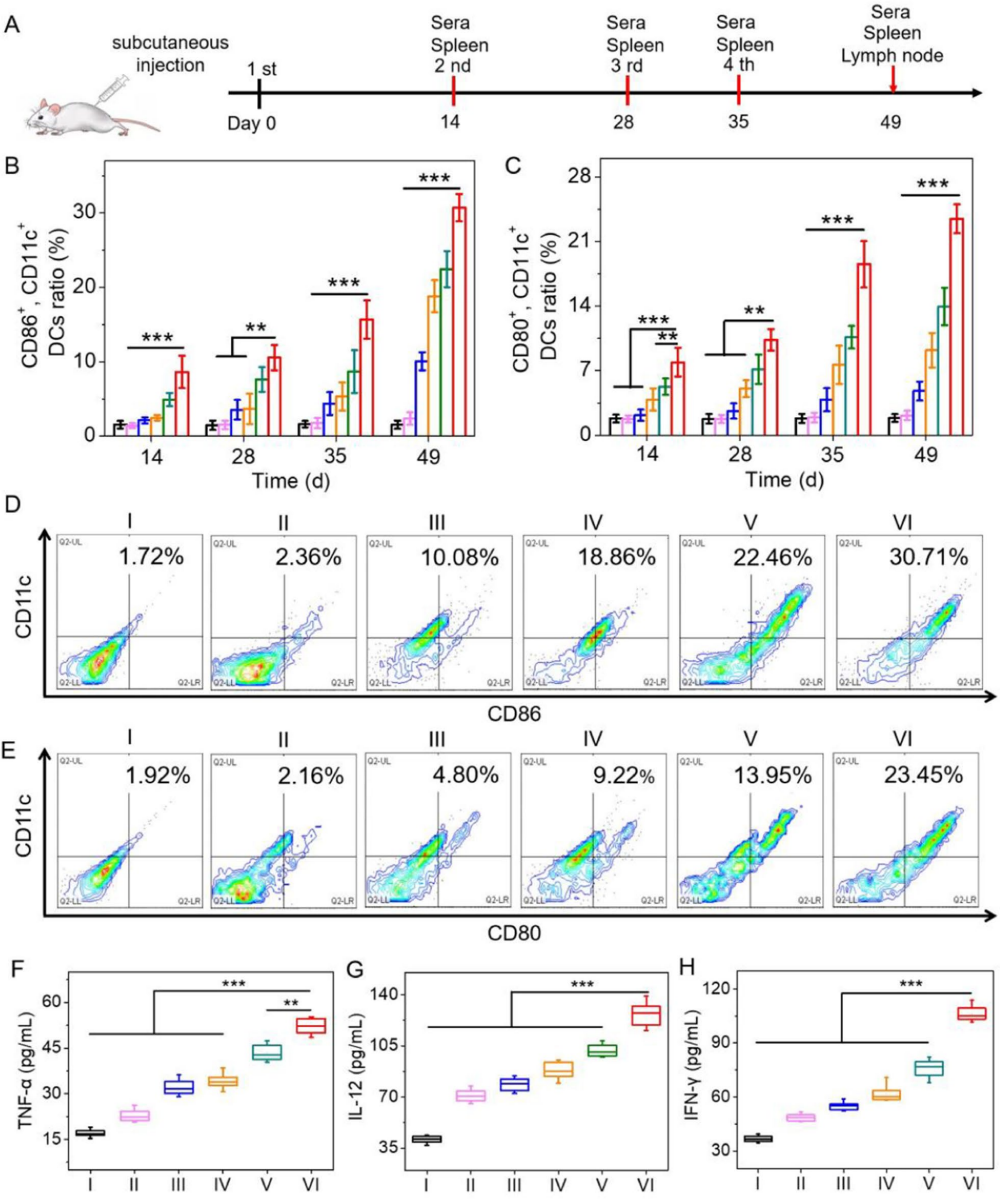
CMR-PLGA/p54 NVs-elicited DCs maturation in vivo. **A** Schematic illustration showing the immunization protocol. **B**, **C** Statistic analysis of activated splenic DCs at different time points. **D**, **E** Flow cytometry analysis of activated splenic DCs collected on day 49. The DCs were collected from mice treated as described in (**A**). **F**–**H** The secretion level of TNF-α, IL-12 and IFN-γ from activated splenic DCs after various immunization. (I: PBS; II: free p54; III: p54 + FA; IV: PLGA/p54 NVs; V: MR-PLGA/p54 NVs; VI: CMR-PLGA/p54 NVs)

To evaluate the protective immunity of NVs against virus infection in vivo, ASFV protein p54 was served as antigen to prepare the CMR-PLGA/p54 NVs (Additional file 1: Fig. S2). ASF is an acute, contagious, lethal infectious disease caused by ASFV, thus leading to the ~ 100% mortality rate and serious economic loss [49–51]. Therefore, it is imperative to develop vaccines to improve the protective immunity of animals against ASFV infection. DCs maturation has critical impact on eliciting the generation of antibody and T cell responses. To assess the DCs maturation in vivo, mice were immunized with different groups, and the DCs were further isolated from spleen on different time points (Fig. 5A). As shown in Fig. 5B–E, CMR-PLGA/p54 NVs induced higher level of stimulatory marker such as CD80 and CD86 compared with other groups, indicating that CMR-PLGA/p54 NVs could greatly promote splenic DCs maturation in vivo. Alternatively, the supernatants from splenic lysates were collected and measured by ELISA to investigate the level of cytokines including TNF-α, IL-12, IFN-γ. In consistence with the results of flow cytometry, the level of cytokines in CMR-PLGA/p54 NVs-treated spleen was higher than that in other groups-treated spleen (Fig. 5F–H, Additional file 1: Fig. S3). The similar results were also noted in LNs, wherein CMR-PLGA/p54 NVs significantly provoked the secretion of TNF-α, IL-12, IFN-γ (Additional file 1: Fig. S4). Collectively, these results indicated that CMR-PLGA NVs could dramatically promote DC maturation through CpG-assisted immune stimulation and mannose-assisted DC targeting.

### In vivo CMR-PLGA/p54 NVs-elicited robust immune responses

**Fig. 6:**
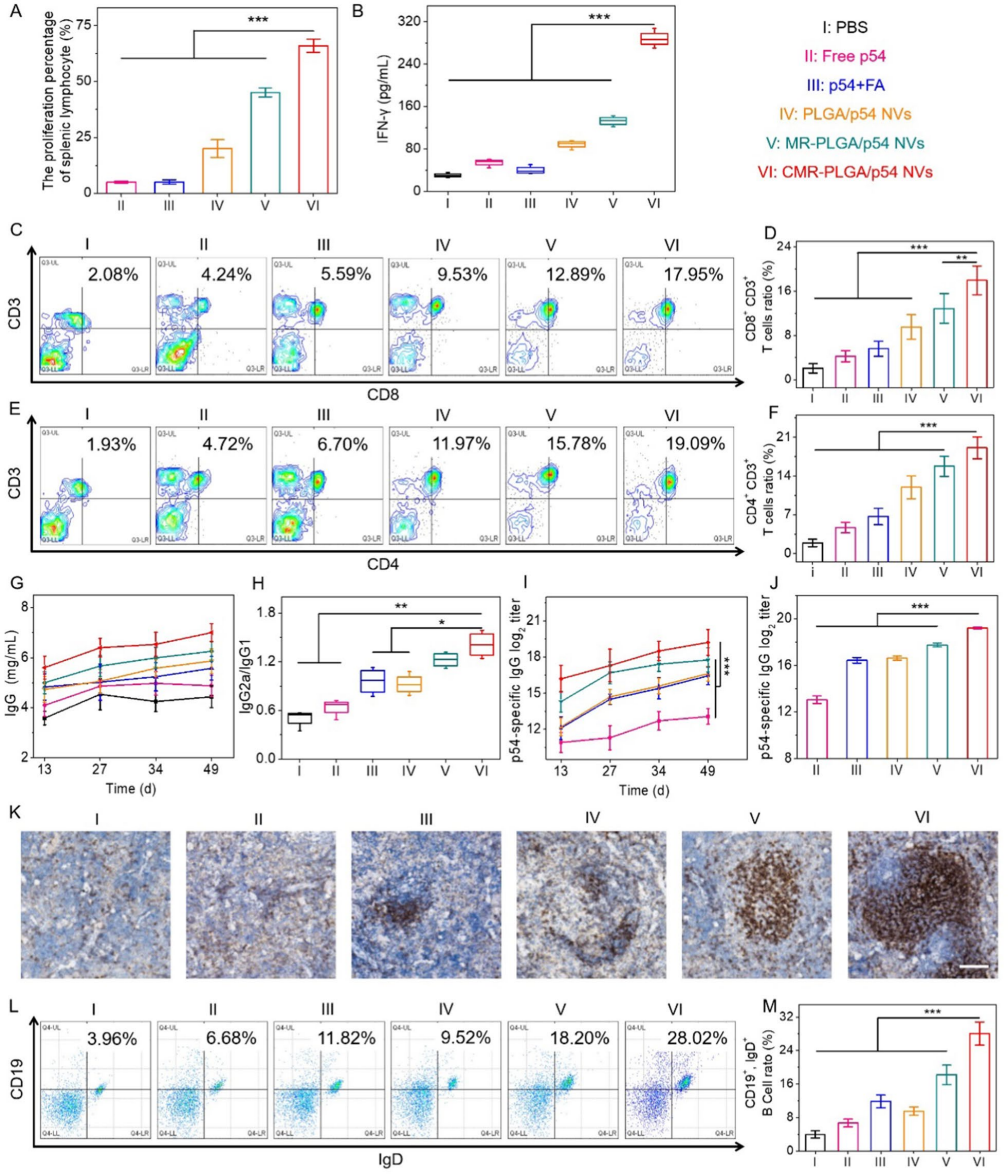
CMR-PLGA/p54 NVs enhanced immune responses in vivo. **A** The proliferation percentage analysis of splenocytes. Splenocytes harvested from immunized mice were re-stimulated with different groups overnight. **B** The IFN-γ content in medium after re-stimulation. Flow cytometry analysis (**C**, **E**) and statistic analysis (**D**, **F**) of activated splenic CD8^+^ and CD4^+^ T cells. The splenic T cells were collected from mice following the immunization as described in Fig. 5A. **G** The level of IgG in serum collected from mice immunized with different groups. **H** The ratio of IgG2a to IgG1 in serum from immunized mice. **I** Changes in specific IgG titers in mice after immunization with different vaccine components. **J** The p54-specific IgG antibody titers in serum measured by ELISA on day 49 (n = 8). **K** Immunohistochemical staining of CD21 in spleen harvested from immunized mice. Bar represents 100 μm. Flow cytometry analysis (**L**) and statistic analysis (**M**) of maturated CD19^+^ IgD^+^ B cells

Based on the remarkable ability of CMR-PLGA/p54 NVs to elicit DCs maturation, we further investigated the immunogenicity of CMR-PLGA/p54 NVs in vivo. Cellular immunity plays an important role on preventing viral infectious diseases. After being immunized with PBS, free p54, free p54 + Freund's adjuvant (FA), PLGA/p54 NVs, MR-PLGA/p54 NVs, and CMR-PLGA/p54 NVs on day 0, 14, 28, 35, spleen was harvested from immunized mice on day 49, homogenized to obtain single-cell suspensions. After being re-stimulated with CMR-PLGA/p54 NVs ex vivo, the proliferation proportion of splenocytes and the IFN-γ content was respectively 13.2-fold, 4.9-fold higher than that in free p54, suggesting that CMR-PLGA/p54 NVs could elicit antigen-specific cellular responses to achieve T cell proliferation (Fig. 6A, B). CD8^+^ T cells are one of critical immune cells that can protect body via killing the invading cells. Hence, the activation of CD8^+^ T cells was further assessed using flow cytometry. As illustrated in Fig. 6C and D, the proportion of CD3^+^ CD8^+^ T cells in mice immunized with PBS significantly increased from 4.24% to 17.95% in CMR-PLGA/p54 NVs immunized mice, which indicated that CMR-PLGA/p54 NVs had the potential to promote the proliferation and activation of CD8^+^ T cells. Alternatively, The CD4^+^ T cells have an important effect on regulating cellular and humoral immunity. After being immunized with CMR-PLGA/p54 NVs, the proportion of CD4^+^ T cells was significantly higher than other groups, indicating CMR-PLGA/p54 NVs-assisted proliferation and activation of CD4^+^ T cells (Fig. 6E, F). Collectively, CMR-PLGA/p54 NVs could activate both CD4^+^ and CD8^+^ T cell responses to elicit robust cellular immunity, which attributed to CMR-PLGA/p54 NVs-elevated spleen accumulation and targeting delivery antigen to DCs.

Alternatively, to investigate the humoral immunity, serum was collected from immunized mice for ELISA measurement. As shown in Fig. 6G, the elevated level of IgG in serum was observed when mice immunized with CMR-PLGA/p54 NVs. Alternatively, the ratio of IgG2a to IgG1 is a marker for Th1 and Th2 immune responses [52]. As shown in Fig. 6H, CMR-PLGA/p54 NVs could elicit higher IgG2a/IgG1 ratio compared with other groups, indicating that CMR-PLGA/p54 NVs favored a Th1-biased immune response. Besides, CMR-PLGA/p54 NVs could dramatically elicit the higher titers of p54-specific IgG than other groups, wherein the titers elevated by CMR-PLGA/p54 NVs were 17.3-fold higher than that induced by free p54 (Fig. 6I, J). The similar results were also obtained in p72 (ASFV antigen), where CMR-PLGA/p72 NVs significantly provoked the titers of p72-specific IgG in serum (Additional file 1: Fig. S5). Besides, B cell maturation and activation of germinal center (GC) in the spleen were further investigated. The GC response in the LNs or spleen is critical for long-lived humoral immunity [53, 54]. Compared with other control groups, higher percentage of CD19^+^ IgD^+^, CD21^+^ GC B cells was shown in the spleen collected from mice immunized with CMR-PLGA/p54 NVs, indicating that CMR-PLGA/p54 NVs also could promote B cell maturation (Fig. 6K, M). Collectively, these results suggested that the CMR-PLGA/p54 NVs could able to elicit robust humoral immune responses.

### In vitro and in vivo safety

**Fig. 7:**
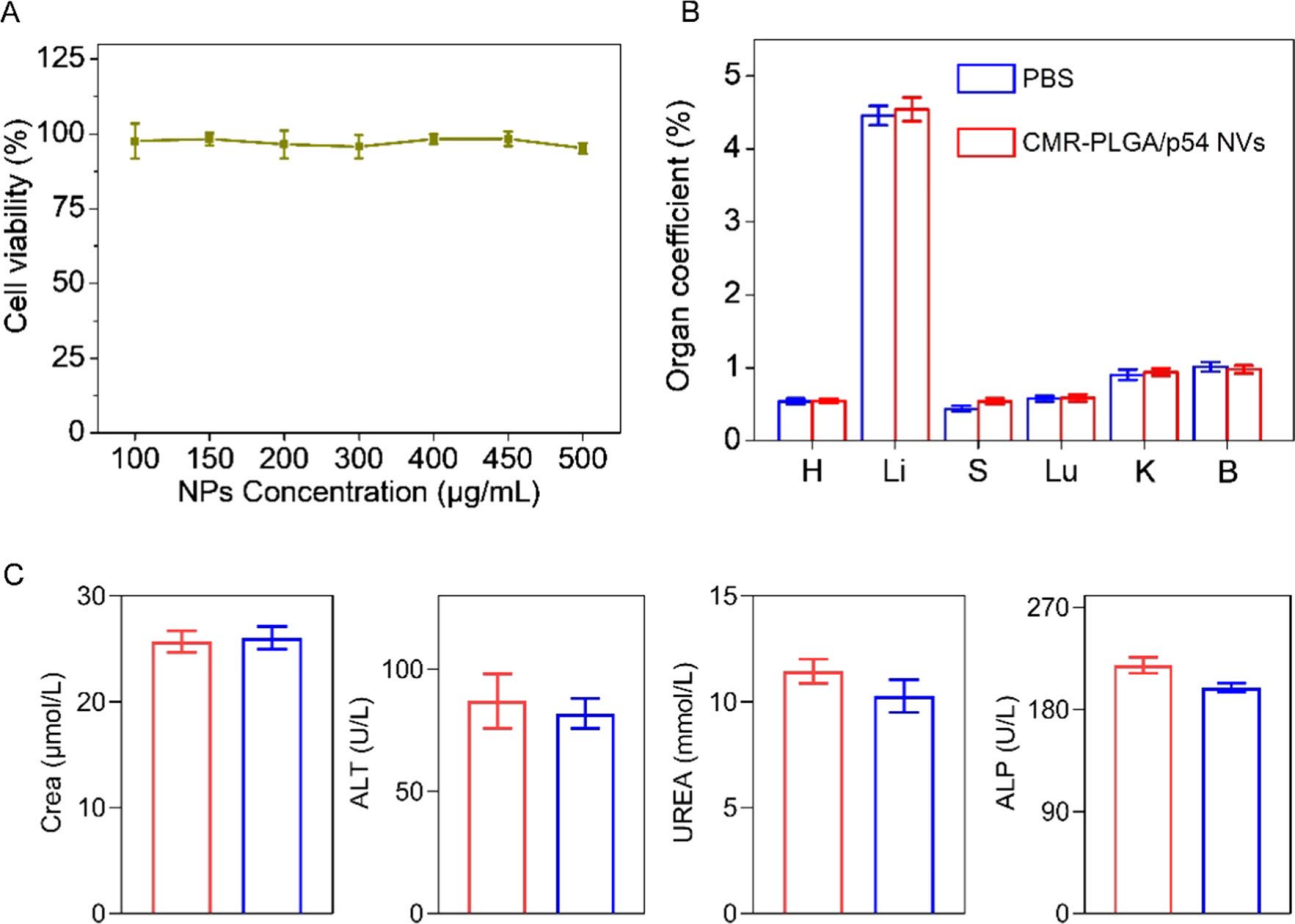
The safety of CMR-PLGA/p54 NVs in vitro and in vivo. **A** Cytotoxicity of MR-PLGA/OVA NVs to BMDCs following 12 h incubation. The organ coefficient (**B**) and hematological assessment (**C**) of mice on day 14 after immunization with CMR-LPGA/p54 NVs. (H: Heart; Li: liver; S: Spleen; Lu: Lung; K: Kidney; B:Brain)

To evaluate the biocompatibility of MR-PLGA NPs, MR-PLGA NPs were incubated with BMDCs for 12 h. The cell viability remained more than 90% at the MR-PLGA NPs concentration up to 500 μg/mL, substantiating the good biocompatibility of CMR-PLGA NPs (Fig. 7A).

During the immunization period, the body weight of mice immunized with free p54 + FA began to decrease on day 28, and the nodules, hair loss were observed at the injection site. In comparison, NVs-immunized mice possessed the gradually increased body weight and minimal side effect (Additional file 1: Fig. S6). Histological examination measured by H&E staining further demonstrated that all NVs immunized mice had negligible damage of major organs (Additional file 1: Fig. S7). After 14 days post immunization of CMR-PLGA/p54 NVs, the organ coefficients (Fig. 7B), alanine creatinine (CRE), aminotransferase (ALT), urea nitrogen (UREA) and alkaline phosphatase (ALP) levels were not significantly increased (Fig. 7C). These results collectively indicated the in vivo desired safety of CMR-PLGA/p54 NVs.

## Conclusions

In conclusion, we have developed p54 (viral antigen) and CpG (a TLR-9 agonist) co-loaded PLGA NVs coated with CpG and mannose co-modified RBC membrane, which could be served as an effective and safe manner to boost protective immunity against virus infection. Based on the splenic accumulation ability of RBC and DC targeting capacity of mannose, antigen could be effectively delivered to the splenic DCs, thus elevating the humoral and cellular immune responses. Excitingly, high levels of DCs maturation and cytokines, remarkable titers of p54 specific IgG, high percentage of CD4^+^ and CD8^+^ T cells, CD19^+^ IgD^+^ B cells, and negligible side effect were all noted in mice immunized with CMR-PLGA/p54 NVs. In addition, CMR-PLGA NPs possessed simple preparation method and could be stored in lyophilized powder for a long time, thus increasing the possibility of clinical translation. Therefore, this study provides a universal, effective, and safe approach for combating viral infectious diseases.

## Supplementary Information

**Additional file 1:** **Figure S1.** Alternation of particle size of PLGA/OVA NVs and MR-PLGA/OVA NVs after incubation with PBS or 1640 containing FBS. **Figure S2.** Expression of ASF antigen. **Figure S3**. Serum level of TNF-α, IL-12 and IFN-γ from activated splenic DCs at different time points. **Figure S4**. The TNF-α, IL-12 and IFN-γlevels in lymph node determined by ELISA. **Figure S5.** Changes in specific IgG titers in mice after immunization with different groups. The p72-specific IgG antibody titers in serum measured by ELISA on day 49. **Figure S6.** Image of mice immunized as described in Fig. 5. Body weight changes of mice. **Figure S7**. H&E staining of major organ sections harvested from mice immunized as described.

## Data Availability

The datasets used and/or analyzed during the current study are available from the corresponding author on reasonable request.
