# Supplementary material for: Splenic-targeting biomimetic nanovaccine for elevating protective immunity against virus infection
Source: J Nanobiotechnology. 2022 Dec 3;20:514. doi: 10.1186/s12951-022-01730-0 (PMC9719655; doi:10.1186/s12951-022-01730-0)
Supplement: Supplementary file 1 — Additional file 1: Figure S1. Alternation of particle size of PLGA/OVA NVs and MR-PLGA/OVA NVs after incubation with PBS or 1640 containing FBS. Figure S2. Expression of ASF antigen. Figure S3. Serum level of TNF-α, IL-12 and IFN-γ from activated splenic DCs at different time points. Figure S4. The TNF-α, IL-12 and IFN-γlevels in lymph node determined by ELISA. Figure S5. Changes in specific IgG titers in mice after immunization with different groups. The p72-specific IgG antibody titers in serum measured by ELISA on day 49. Figure S6. Image of mice immunized as described in Fig. 5. Body weight changes of mice. Figure S7. H&E staining of major organ sections harvested from mice immunized as described. [file 12951_2022_1730_MOESM1_ESM.docx]

Splenic-targeting biomimetic nanovaccine for elevating protective immunity against virus infection

Jian Huo^#^, Aangke Zhang^#^, Shuqi Wang^#^, Hanghang Cheng, Daopeng Fan, Ran Huang, Yanan Wang, Bo Wan, Gaiping Zhang, Hua He*

College of Veterinary Medicine, International Joint Research Center of National Animal Immunology, Henan Engineering Laboratory of Animal Biological Products, Longhu Laboratory, Henan Agricultural University, Zhengzhou 450046, China

^#^These authors contributed equally to this work.

*Corresponding author: Hua He [hehua1123@126.com](mailto:hehua1123@126.com) (Email).

**Methods**

**Materials and animals**

Poly (D, L-lactide-co-glycolide) (PLGA, 50:50, Mw = 38000-54000 Da) and polyvinyl alcohol (PVA) were purchased from Sigma-Aldrich (St Louis, MO, USA). BCA kit and MTT were obtained from Beyotime Biotechnology (Shanghai, China). Ovalbumin (OVA), FITC, and Cy5.5 were purchased from Aladdin (Shanghai, China). Chol-CpG was purchased from Sangon Biotech (Shanghai, China). Freund's Complete Adjuvant and Freund's incomplete adjuvant were purchased from from Sigma-Aldrich (St Louis, MO, USA). Anti-TNF-α, anti-IFN-γ, anti-IL-12, anti-IgG1 and anti-IgG2a ELISA kits and anti-SIINFEKL-PE were purchased from Thermo Fisher Scientific (Waltham Mass, USA). Anti-CD80-FITC, anti-CD86-FITC, anti-CD11c-APC, anti-CD3-APC, anti-CD4-FITC, anti-CD8-FITC were all purchased from Abcam (Cambridge Science Park, UK). Anti-MHC-II-FITC, anti-CD19-APC, anti-IgD-FITC were purchased from Biolegend (California, USA). All solvents were purchased from Sinopharm Chemical Reagent Co. Ltd (Shanghai, China).

Female BALB/c mice (6-8 weeks) were purchased from Animal experimental center of Huaxing (Zhengzhou, China) and housed in clean room. All animal studies were approved by the Institutional Animal Care and Use Committee, Henan Agricultural University.

**Preparation of RBC membrane and ligand-inserted RBC membrane**

The red blood cell (RBC) membrane was extracted by hypotonic hemolysis method [1, 2].{Li, 2022 #6400}{Li, 2022 #6400} The whole blood (~1 mL) was collected from BALB/c mice, and further centrifuged (3500 rpm, 4 ℃) for 10 min to obtain RBCs. After washing with cold PBS, the RBCs was diluted with 0.25 × PBS (40 mL) and further incubated at 4 ℃ for 2 h. Repeated centrifugation (9000 rpm, 4 ℃, 10 min) was done four times. Finally, the RBC membranes were suspended in PBS and lyophilized. To form the CpG and mannose co-inserted RBC membranes, the RBC membrane (50 mg), DSPE-PEG-Man (40 μg), and chol-CpG (15 μg) were dissolved in PBS and the mixture solution further co-incubated at 37 °C for 30 min. After centrifugation (6000 rpm, 4 °C) for 5 min, the precipitation was washed twice with PBS to obtain CpG-mannose-inserted RBC membranes (CpG-Man-RBCm).

**Synthesis of fluorescence-labeled OVA**

To observe and quantify OVA *in vitro* and *in vivo*, OVA was labeled with fluorescent tags. For *in vitro* assay, OVA (10 mg) was dissolved in Na_2_CO_3_ solution (10 mL, pH 9.8, 25 mM), and FITC (200 μL, 1 mg/mL in DMSO) solution was added which was stirred in the dark at room temperature (RT) overnight. The obtained FITC-OVA was dialyzed against DI water (MWCO = 3500 Da) for 3 days and lypophilized. For *in vivo* assay, the Cy5.5-NHS (1 mL, 1 mg/mL in PBS) solution was added to the PBS solution of OVA (10 mg, 5 mL), stirred in the dark at RT for 12 h, and dialyzed against DI water (MWCO = 3500 Da) for 2 days. After lypophilization, the Cy5.5-OVA was obtained.

**The characterization of CMR-PLGA/OVA NVs**

The particle size and zeta potential were measured by dynamic laser scanning (DLS, Malvern Nano ZS), and the morphology was monitored by transmission electron microscopy (TEM). To investigate the serum stability of NVs, PLGA/OVA NVs, MR-PLGA/OVA NVs were diluted with 1640 medium containing 10% FBS and incubated at RT for various time before the measurement of the particle size. The membrane proteins loaded on MR-PLGA/OVA NVs and RBC membranes were analyzed by SDS-PAGE.

To determine the protein loading content (PLC), MR-PLGA/OVA NVs were centrifuged (14500 rpm, 4 °C) for 20 min, the supernatant was collected to measure the protein loading content (PLC) and protein loading efficiency (PLE) of OVA by BCA kit, and calculated as the below:

PLC (%) = × 100

PLE (%) = × 100

***In vitro* protein release**

MR-PLGA/OVA NVs were dissolved in PBS and shaken (220 rpm, 37 ℃). At designated time, the release medium was centrifuged (13000 rpm, 10 min), and the OVA content in the suspernatant was determined by BCA kit.

**Preparation of bone marrow-derived DCs**

Bone marrow-derived DCs (BMDCs) were obtained as previously described.^[3]^ Female BALB/c mice were sacrificed and soaked in alcohol for 5 min. Tibia and fibula were dissected, and the bone marrow cavity was repeatedly rinsed with cold PBS. After centrifugation (1800 rpm, 4 °C) for 5 min, cells were suspended with 2 mL red blood cell lysate and reacted for 2 min. PBS (20 mL) was added to stop the reaction and centrifuged (1800 rpm, 4 °C) for 5 min. The precipitates were collected, suspended in a medium containing IL-4 (5 ng/mL) and GM-CSF (5 ng/mL), and cultured for 5-7 days.

***In vitro* cytotoxicity**

To evaluate the cytotoxicity of NVs, BMDCs were seeded on 96-well plates at 3×10^4^ cells/well and cultured for 3 h. The MR-PLGA/OVA NVs were added at various concentrations of NVs, and cells were further co-incubated for 12 h before viability assessment by the MTT assay.

***In vivo* biodistribution**

Female BALB/c mice were subcutaneously injected with free Cy5.5-OVA, PLGA/Cy5.5-OVA NVs, and MR-PLGA/Cy5.5-OVA NVs at 50 µg Cy5.5-OVA/mouse (n = 3). Fluorescence imaging was performed at various time points post subcutaneously injection by an *in vivo* imaging system (IVIS^®^ Lumina Ⅲ, PerkinElmer, USA). In a parallel assay, mice were sacrificed at 72 h post injection. The major organs including heart, liver, spleen, lung, kidney, lymph nodes were harvested, and imaged using in vivo imaging system.

***In vivo* safety**

BALB/c mice were subcutaneously immunized with PBS and CMR-PLGA/p54 NVs (30 μg p54/mouse). To evaluate the acute toxic effects, liver, kidney, brain, lung, spleen and heart were collected on day 14 to calculate the organ coefficients (The organ weight/body weight). Besides, blood was also collected to detect aspartate aminotransferase (AST), alanine creatinine (CRE), aminotransferase (ALT), urea nitrogen (urea) and alkaline phosphatase (ALP) levels.

**Statistical analysis**

Data could be expressed as mean ± standard deviation. Student’s t-test was used for statistical comparisons. The differences were set to be significant at *p < 0.05 and very significant at **p < 0.01, ***p < 0.001.

**References**

1. Li J, et al. Red Blood Cell-Mimic Nanocatalyst Triggering Radical Storm to Augment Cancer Immunotherapy. Nanomicro Lett. 2022, 14**:**57-71.

2. Dai J, et al. Red blood cell membrane-camouflaged nanoparticles loaded with AIEgen and Poly(I : C) for enhanced tumoral photodynamic-immunotherapy. Natl. Sci. Rev. 2021, 8**:** 039-053.

3. Liu X, et al. A cell-penetrating peptide-assisted nanovaccine promotes antigen cross-presentation and anti-tumor immune response. Biomater Sci. 2019, 7**:**5516-5527.


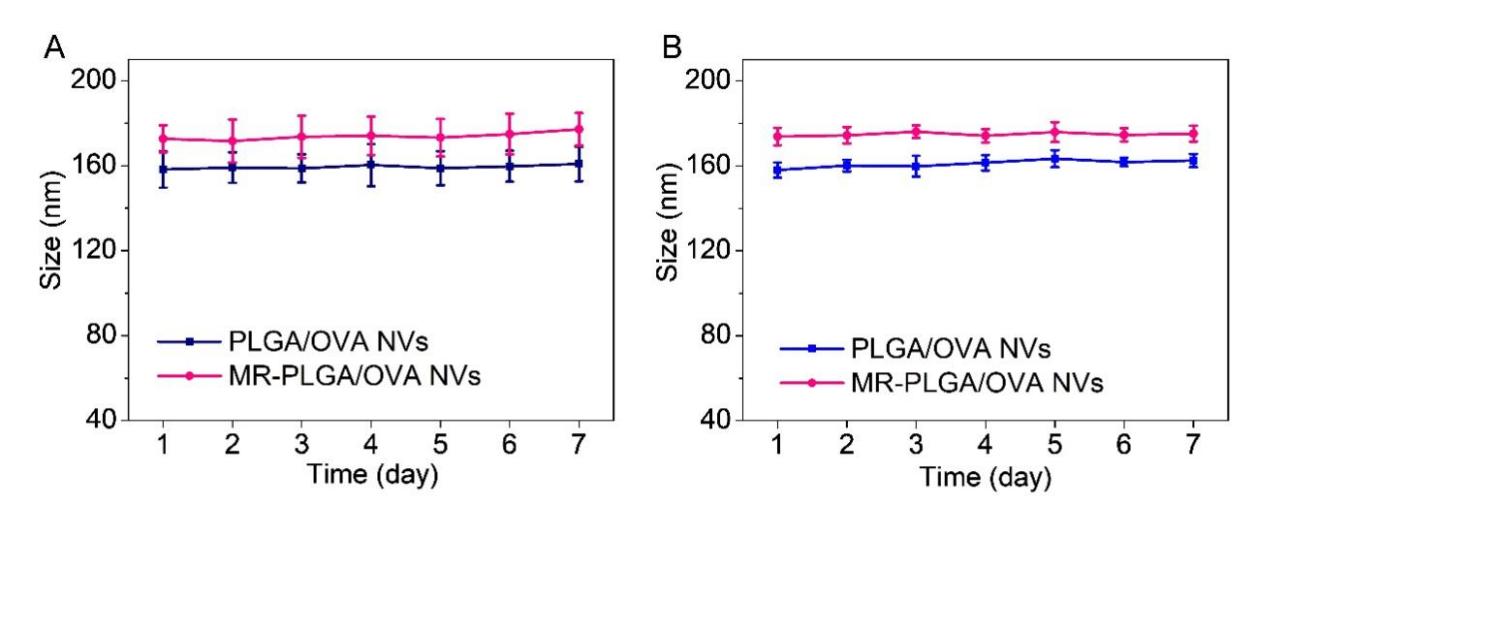


**Figure S1**. Alternation of particle size of PLGA/OVA NVs and MR-PLGA/OVA NVs after incubation with PBS (A) or 1640 containing FBS (B) (n = 3).


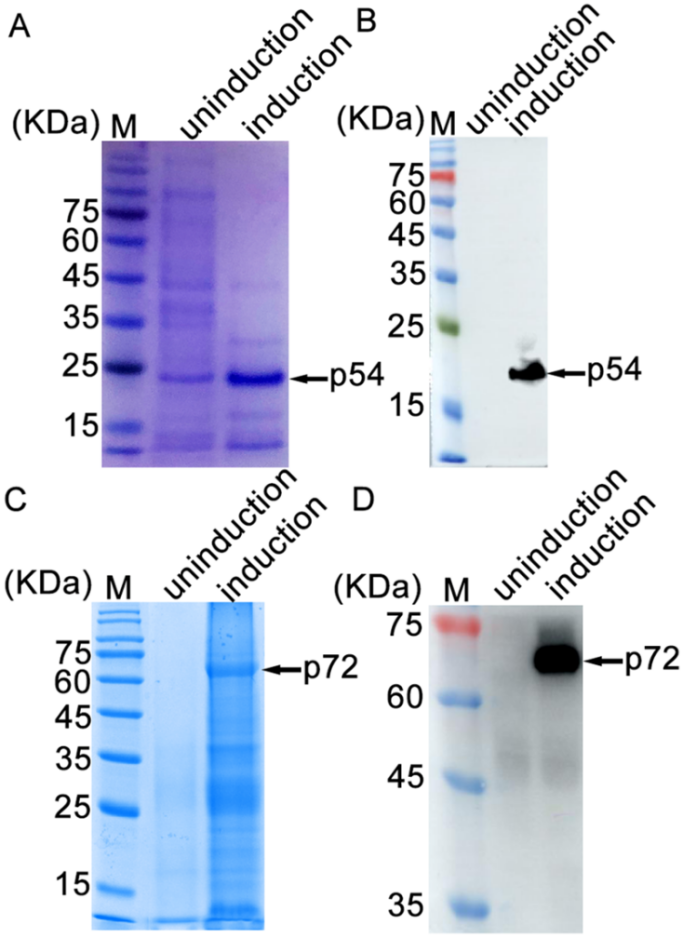


**Figure S2.** Expression of ASF antigen. (A, B) SDS-PAGE and immunoblot of p54 protein. (C, D) SDS-PAGE and immunoblot of p72 protein.


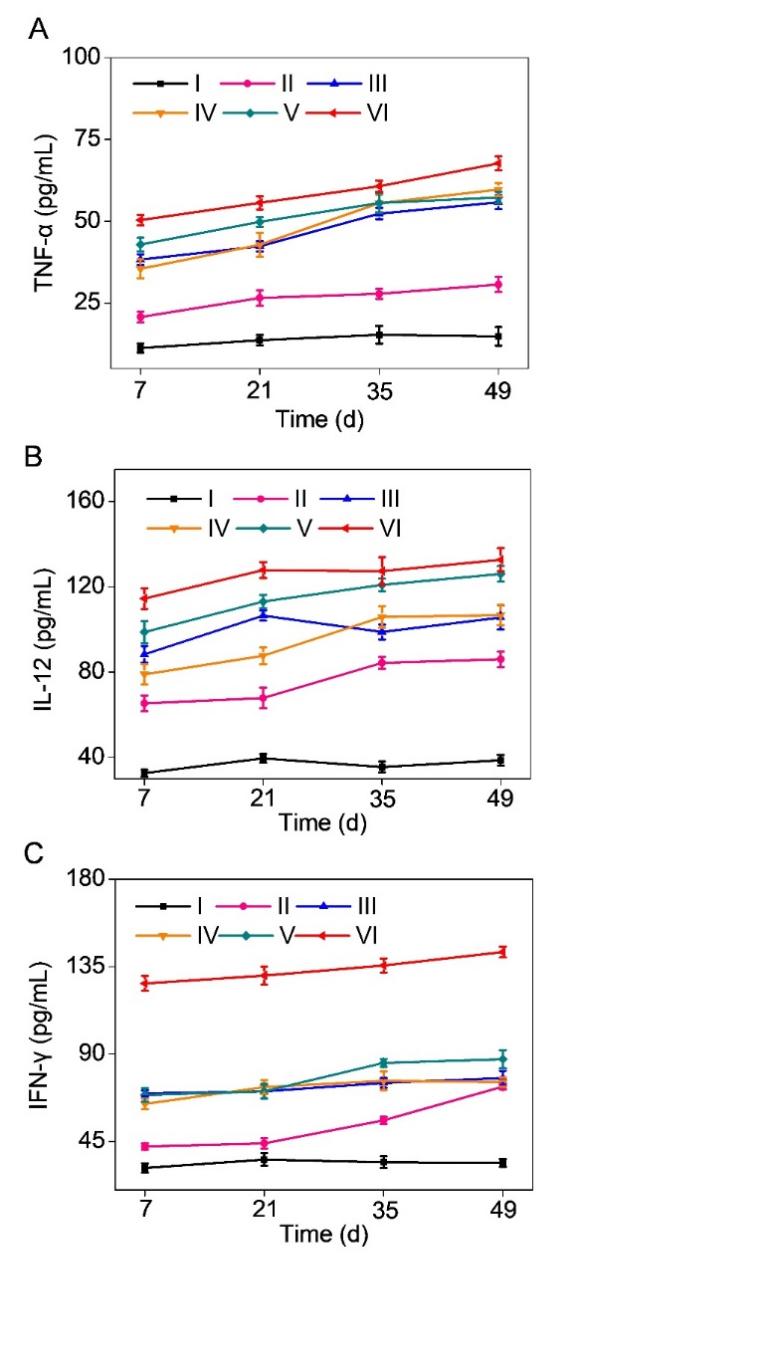


**Figure S3**. Serum level of TNF-α (A), IL-12 (B) and IFN-γ (C) from activated splenic DCs at different time points. Spleen was harvested from mice immunized as described in Figure 5. (I: PBS; II: free p54; III: p54+FA; IV: PLGA/p54 NVs; V: MR-PLGA/p54 NVs; VI: CMR-PLGA/p54 NVs)


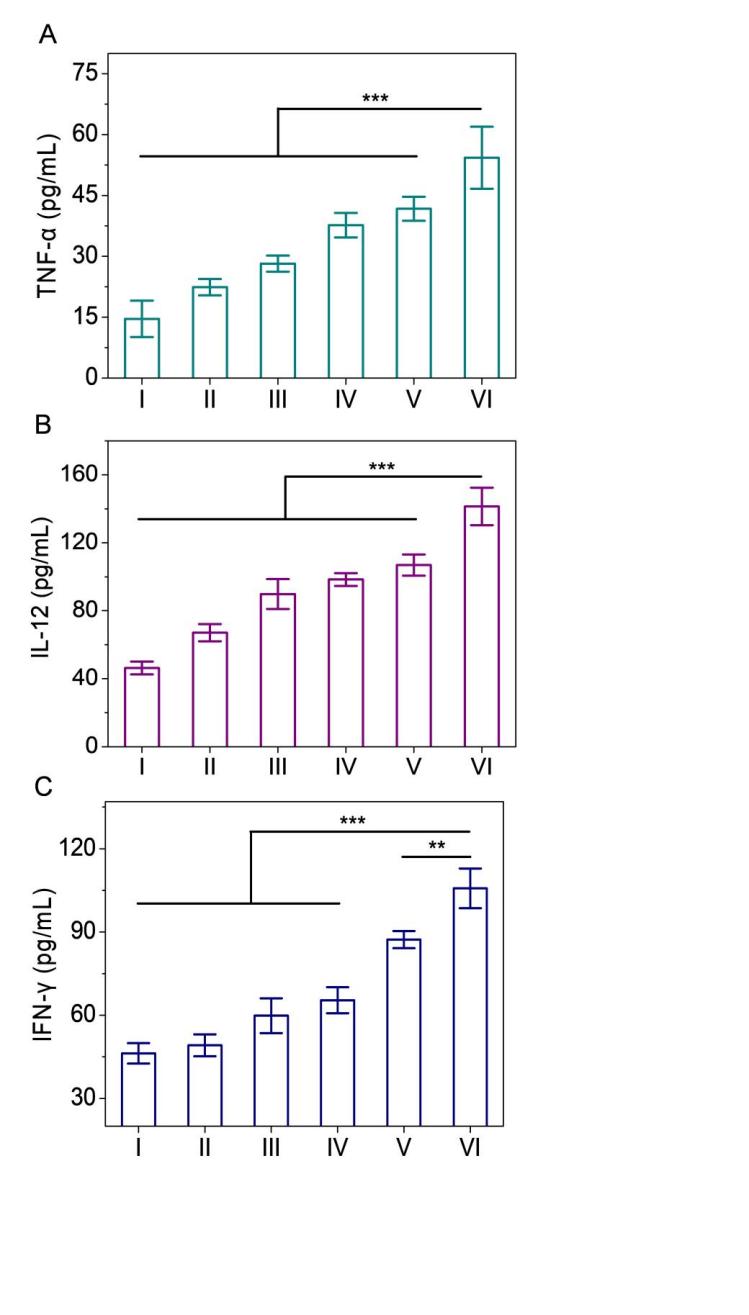


**Figure S4**. The TNF-α (A), IL-12 (B) and IFN-γ (C) levels in lymph node determined by ELISA. Lymph node was harvested from mice immunized as described in Figure 5. (I: PBS; II: free p54; III: p54+FA; IV: PLGA/p54 NVs; V: MR-PLGA/p54 NVs; VI: CMR-PLGA/p54 NVs)


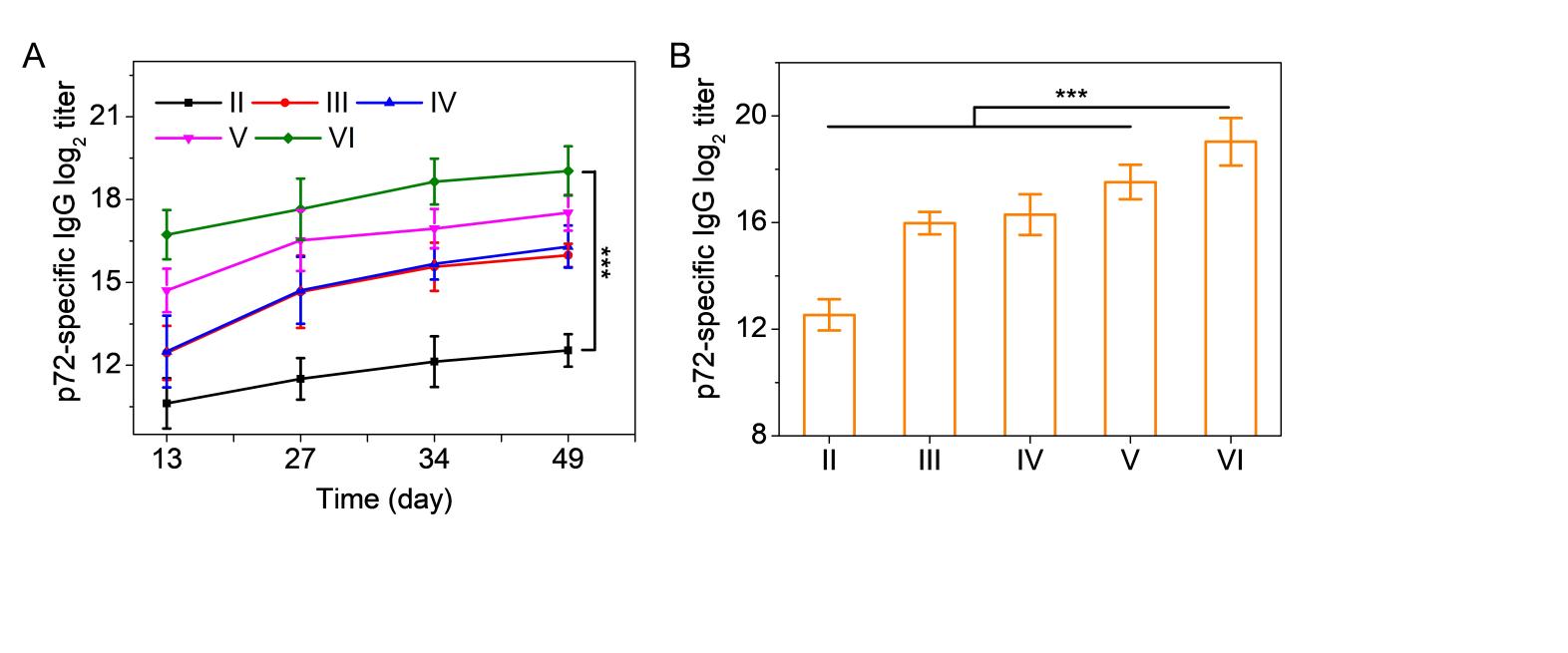


**Figure S5**. (A) Changes in specific IgG titers in mice after immunization with different groups. (B) The p72-specific IgG antibody titers in serum measured by ELISA on day 49 (n = 8). (I: PBS; II: free p72; III: p72+FA, IV: PLGA/p72 NVs; V: MR-PLGA/p72 NVs; VI: CMR-PLGA/p72 NVs)


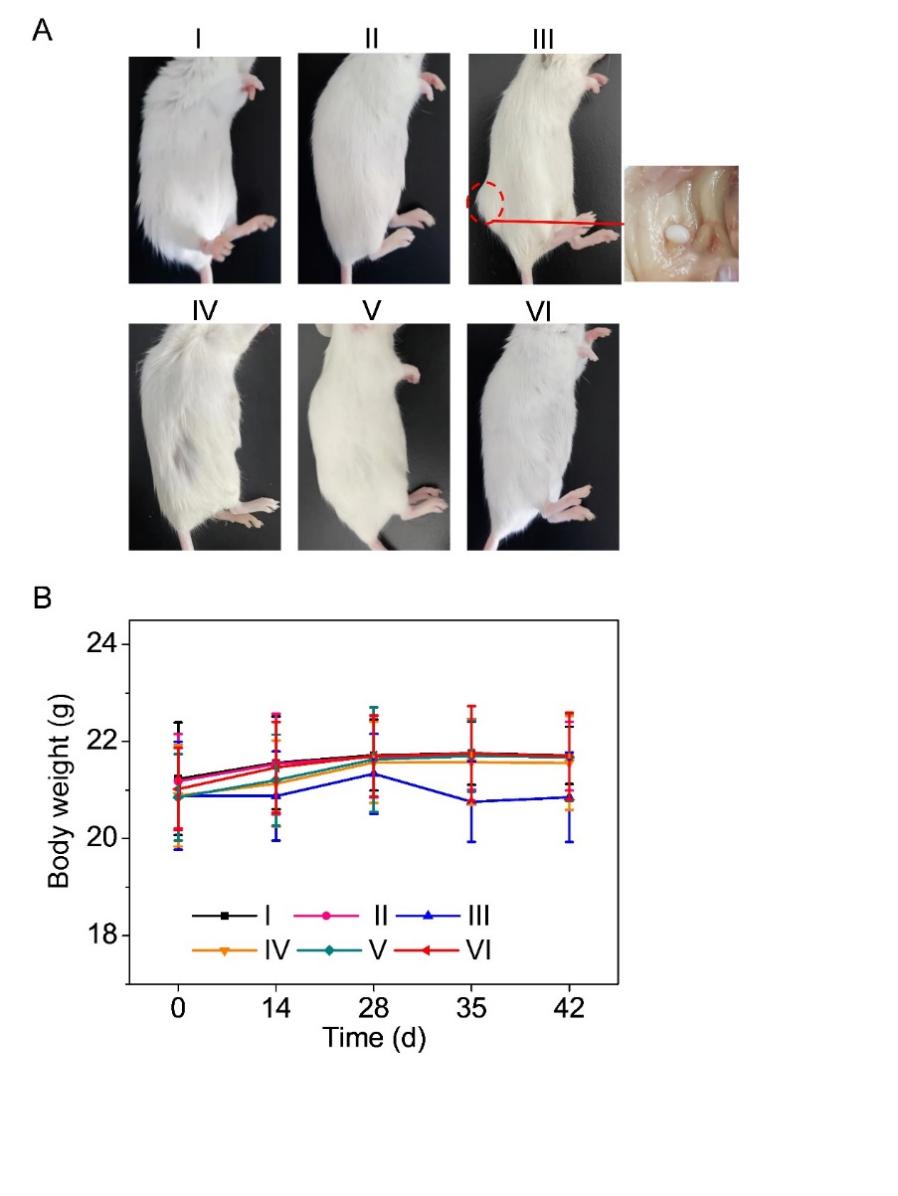


**Figure S6**. (A) Image of mice immunized as described in Figure 5. (B) Body weight changes of mice. (I: PBS; II: free p54; III: p54+FA; VI: PLGA/p54 NVs; V: MR-PLGA/p54 NVs; VI: CMR-PLGA/p54 NVs)


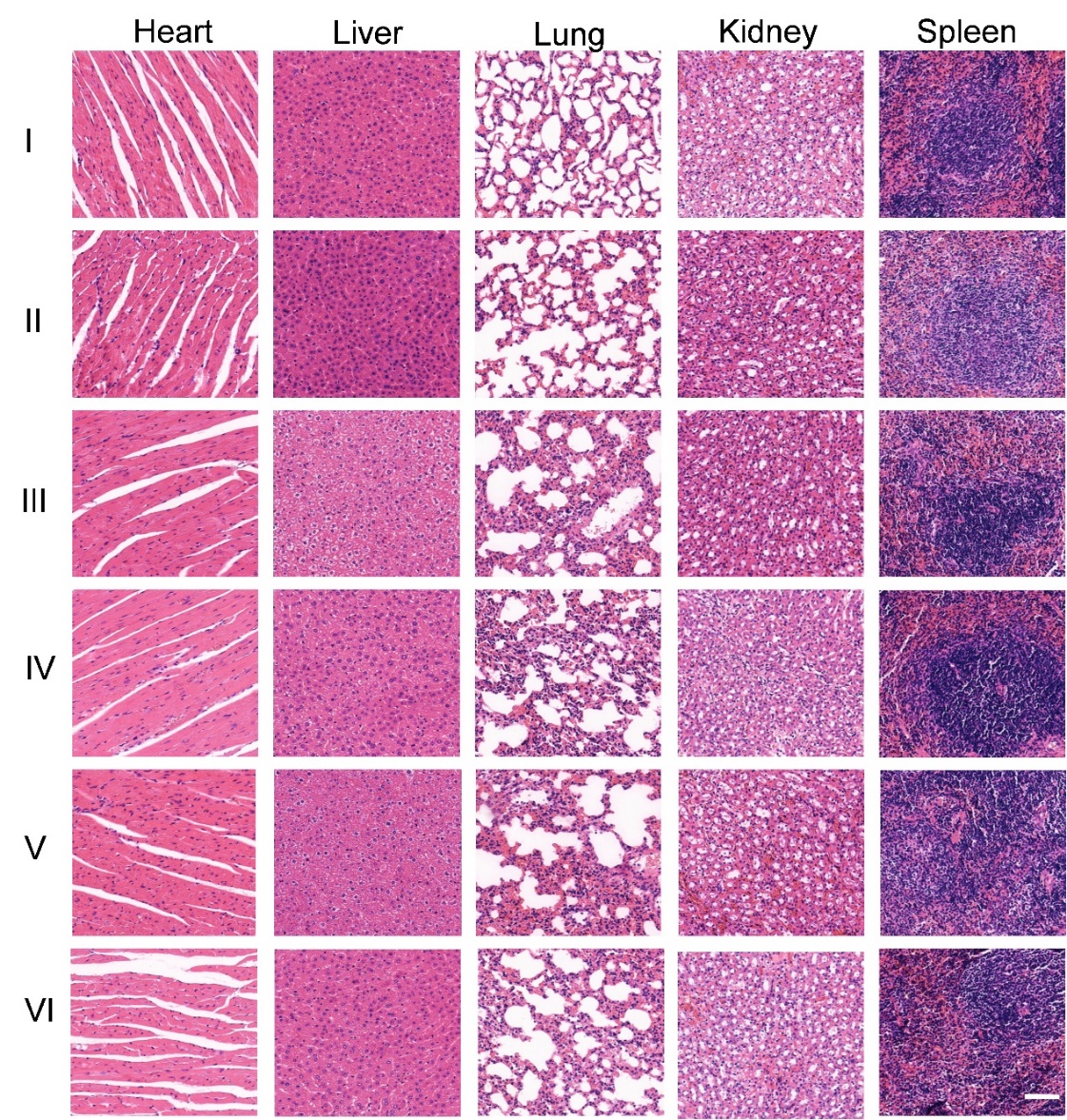


**Figure S7**. H&E staining of major organ sections harvested from mice immunized as described in Figure 5 on day 49. Bar represents 40 μm. (I: PBS; II: free p54; III: p54+FA, IV: PLGA/p54 NVs; V: MR-PLGA/p54 NVs; VI: CMR-PLGA/p54 NVs)
